# Green method for improving performance attributes of wool fibres using immobilized proteolytic thermozyme

**DOI:** 10.1007/s13205-022-03323-y

**Published:** 2022-09-02

**Authors:** Sanaa K. Gomaa, Rania A. Zaki, Marwa I. Wahba, Marwa Abou Taleb, Heba A. El-Refai, Asmaa F. El-Fiky, Hosam El-Sayed

**Affiliations:** 1 Chemistry of Natural and Microbial Products Department, National Research Centre, Giza, 12622 Dokki Egypt; 2 Proteinic and Man-made Fibres Department, Textile Research and Technology Institute, National Research Centre, Giza, 12622 Dokki Egypt; 3 Centre of Scientific Excellence-Group of Advanced Materials and Nanotechnology, National Research Centre, Giza, 12622 Dokki Egypt

**Keywords:** Wool, Felting shrinkage, Dyeing, Thermophilic protease, Sericin, Immobilization

## Abstract

Wool has the tendency to turn into felt during agitation in washing machines. Thus, a benign non-polluting method for the production of machine-washable wool was developed herein. Initially, a proteolytic bacteria was isolated from hot region soil. The bacterial isolate was identified as *Bacillus safensis* FO-36bMZ836779 according to the 16S rRNA gene sequencing. Afterwards, the extracellular protease produced by this isolate was covalently immobilized in order to enhance its stability under non-ambient conditions which are usually adopted in industrial sectors like textile industries. Sericin, which is usually discharged into degumming effluent of natural silk, was utilized to prepare the immobilization carrier. Box–Behnken design was adopted in order to hone the preparation of the sericin–polyethylene–imine–glutaraldehyde activated agar carrier. The pH and temperature profiles of the free and immobilized proteases were compared. Later, wool fibres were bio-treated with both the free and the immobilized enzymes. The effect of process conditions on the resistance of the bio-finished wool to felting was investigated. The alteration in the fibre morphology was monitored using SEM. Amino acid analysis and alkali solubility tests were adopted to assign any change in the chemical structure of the bio-treated wool. The influence of bio-treatment of wool on its inherent properties was assigned. Results revealed that bio-treatment of wool with the said enzyme led to production of machine-washable wool without severe deterioration in the fibres’ properties. In an energy- and water-consuming process, the hot solution from bio-treatment bath was used successfully in dyeing of wool.

**Supplementary Information:**

The online version contains supplementary material available at 10.1007/s13205-022-03323-y.

## Introduction

Enzymes are specific biocatalysts that bring about biochemical metabolic processes of the cells. Microbial proteases are among the most widely used classes of industrial enzymes that show specificity and selectivity, that are essentially used in different industrial sectors including textiles, detergents, leather and others. Proteolytic enzymes can catalyse the hydrolysis of peptide bonds along protein macromolecules (Kieliszek et al. 2021). Proteases are categorized into the following two main groups: the endopeptidases, which rupture internal peptide bonds, and exopeptidases, which break C- or N- terminal peptide bonds (Aruna et al. 2014). *Bacillus* sp. is one of the best protease-producing microorganisms that display broad substrate specificity, simple downstream purification, short fermentation time, and significant stability and activity (Haddar et al. 2009).

The immobilization of industrial enzymes is highly favorable as it facilitates the partitioning betwixt the enzymes and their reaction products. The immobilization of enzymes gives the opportunity to recover and reuse these enzymes in industrial processes. Immobilization of enzymes also leads to enhanced stability towards high temperature, pH variation, and other extreme operating conditions (Park et al. 2002).

Within the past 2 decades, different classes of enzymes have been utilized to improve some properties of wool (El-Gabry et al. 2008; Haggag et al. 2013). The currently used chemicals in textile industry have many disadvantages, the most important of which is the discharge of many pollutants into the effluent causing environmental problems (Aruna et al. 2014; El-Sayed et al. 2021; Kantouch et al. 2011b; Mowafi et al. 2014). By virtue of their ecofriendly characteristics together with easy isolation from the micro-organisms, enzymes would replace aggressive chemicals usually used in wet processing of wool (Kantouch et al. 2005; Paranthaman et al. 2009). Bio-treatment of enzymes was successfully carried out in some wet processes including desizing, scouring, and shrink-proofing (El-Fiky et al. 2021; El-Sayed et al. 2010a; Kumar et al. 2021). Being bio-degradable, enzymes are not a source of pollutants if utilized in wet processing of textiles (Srilakshmi et al. 2015).

The use of natural fibres in the clothing field is a customer-demand by virtue of their outstanding comfort and appearance attributes (Ahmed and Mondal 2021; Fu et al. 2015). Wool fibres’ quality is affected by the environment in which the sheep is raised and the amount of lipids present on the epicuticle surface. Processing of textile fibres, like wool, involves extensive wet treatments to improve their performance attributes including softness, anti-pilling and dimensional stability (Abou Taleb et al. 2018; El-Newashy et al. 2019; El-Sayed 2021; Kantouch et al. 2011a). These treatments bring about some environmental concerns such as increased chemical oxygen demand (COD) of the drained water as well as discharge of absorbable organo-halogens into the effluent (Abou Taleb et al. 2020a; El‐Sayed and El‐Khatib 2005; Mowafi et al. 2018). On the other hand, wet processing of wool consumes a lot of energy, water, and time (Huson 2018; Lewis 2013). So many studies, including bio-treatment, have been performed to reduce energy, water, and chemical consumption in textile wet processes (Allam et al. 2020; El-Sayed and El-Hawary 2021; Elshemy et al. 2017; Mowafi et al. 2020, 2022).

Due to unidirectional movement of the scales on the surface of wool, woolen garments tend to turn into felt during mechanical agitation in domestic washing machines. The chemistry and technology of the production of machine-washable wool has been the subject of many investigations (El-Sayed 2021). The chlorine/Hercosett process is the most successful technology adopted for production of machine-washable wool (Hassan and Carr 2019). However, owing to many environmental legislations and the liberation of adsorbable organo-halogen (AOX) from the chlorination of wool, many investigations have been brought about to attain an AOX free felt-proofing process for wool (El‐Sayed and El‐Khatib 2005; Kantouch et al. 2007). Because they are able to digest keratinized proteins, proteases are appropriate candidate for removing the cuticle scales on the surface of wool (El‐Sayed et al. 2001; Erlacher et al. 2006). Partial removal of the cuticle layer together with its superficial lipid barrier from wool fibres by proteolytic action lead to enhancing the dyeability of wool fibre (El‐Sayed et al. 2010c). To avoid any deteriorative effect on the fibre interior, the protease molecules catalysis should be restricted to the fibre surface (El‐Sayed et al. 2002).

Bio-treatment of textile substrates has been greatly increased over the past few decades. Special interest was directed towards extremozymes which have a practical commercial use by virtue of their stability at non-ambient conditions (Ismail et al. 2020; Zeldes et al. 2015). Removal of wool fibre scales to improve its resistance to felting shrinkage was successfully carried out using proteolytic enzymes (Montazer and Ramin 2010; Raja and Thilagavathi 2011).

No research works have been reported for the utilization of immobilized thermophilic protease in felt-proofing of wool fibres. Afterwards, a new thermophile bacteria was isolated from hot spring in Upper Egypt, and its proteolytic activity was enhanced after optimizing the production medium. The thermal stability of the enzyme was enhanced by its immobilization on sericin-containing carrier. The objectives of this work were extended to diminish energy and water consumption during wet processing of wool by conducting both bio-finishing and dyeing of wool in the same bath in two successive steps.

## Experimental

### Materials

Australian merino wool tops were purchased from Misr Company for Spinning and Weaving, Egypt. The mean fibre diameter of the used fibre was 20.2 µm. The acid dye, C.I. Acid Blue 203, was kindly supplied by Egypt Colors Company, Cairo, Egypt. Absolute ethanol and nutrient agar were provided by Fluka Switzerland. Agar was purchased from SD Fine chemicals, Mumbai. Glucose, magnesium sulphate, disodium hydrogen phosphate and sodium dihydrogen phosphate were purchased from BDH chemicals LTD, England. Folin’s reagent was supplied by Loba Chemie. All other used chemicals were of laboratory grade and used as they were supplied. Sericin was extracted from raw silk according to Oh et al. (2011).

### Methods

#### Collection of samples and their characterization

Water probes were collected from hot springs and incubated in sterile thermal glass vessels. Hot springs (Oyoun Mossa) in Egypt and also Red Sea coast were selected for samples collection. Immediately, the collected water samples were used for enrichment in nutrient broth at 55 °C. One-day enrichment culture was streaked on nutrient agar to obtain separate colonies (Mohammad et al. 2017).

Other samples of soil were collected from soil in El Menia, Upper Egypt region at temperature between 45 and 50 °C. Enrichment culture technique was applied (Bodour et al. 2003). One gram of soil samples was serially diluted to 10^–4^ in distilled water, shaken and cultured in nutrient agar medium containing fluconazole 150 mg as an antifungal. The plates were incubated at 45 °C for 24 h. The pure bacterial isolates were obtained and stored at 4 °C with monthly subculture maintenance. The isolated bacteria were screened for the production of protease enzyme. The promising isolated strain was identified using polymerase chain reaction 16S rDNA and electrophoresis analysis and registered in Genbank.

#### Molecular identification

##### DNA extraction

The Gene JET Genomic DNA purification kit 98 (Thermo Scientific # k0721) was adopted for extraction of Genomic DNA of the bacterial isolates.

##### PCR amplification and sequencing of 16S rRNA gene

Partial amplification of the 16S rRNA fragments was carried out by PCR using Maxima Hot Start PCR Master Mix (Thermo K1051) in Sigma Company of Scientific Services, Egypt (www.sigma-co-eg.com). For phylogenetic analysis, the assigned sequences were compared with the sequences deposited in the National Center for Biotechnology Information (NCBI) GenBank database (www.ncbi.nlm.nih.gov) by BLAST search.

#### Cultivation conditions and crude enzyme extraction

Isolated bacteria were first inoculated in liquid nutrient broth medium; after 48 h, 10% volume of the liquid medium was transferred to the production medium using 100 mL of the production medium in a 250-mL flask. The production medium comprised NaCl, 0.5 g/L; K_2_HPO_4_, 0.3 g/L; KH_2_PO_4_, 0.4 g/L; wool, 10 g/L; and the pH was adjusted at 7.0–7.2 using 1 M NaOH and HCl. The cultivated media were shaken (200 rpm) for 5 days at 45 °C. The supernatants were separated by centrifugation (12,000 rpm) at 4 °C for 30 min. The different supernatants which contain the crude enzymes were utilized in assay and analysis of protease enzyme (Hassan et al. 2013).

#### Protein determination

The method of Lowry et al. was adopted to determine the protein content (Lowry et al. 1951).

#### Protease assay

A modified method of that reported by Tsuchida et al. was adopted for quantitative protease activity in the culture filtrate by using casein as the substrate (Tsuchida et al. 1986). Overall, 100 μL of enzyme solution was added to 900 μL of substrate solution [2 mg/mL w/v of casein in 10 mmol/L Tris–HCl buffer (pH 7.0)]. The mixture was incubated at 55 °C for 30 min. Reaction was terminated by the addition of 1 mL of 10% (w/v) trichloroacetic acid, and then the reaction mixture was allowed to stand in ice bath for 15 min to precipitate the insoluble proteins. Finally, the solution was mixed with 5 mL of sodium carbonate and 0.5 mL of Folin's reagent for 25 min and the absorbance value was determined in 750 nm. All assays were carried out in triplicate. One protease unit is defined as the amount of enzyme that releases 1 μg of a tyrosine mL/min under the aforementioned assay conditions. The specific activity is expressed in the units of enzyme activity mg/protein.

#### Effect of incubation periods on protease production

The production medium was inoculated with 10 mL of the spore suspension for 1, 2, 3, 4, 5 and 6 days with continuous shaking at 150 rpm at 45 °C, and; thereafter the analyses were carried out at the end of each incubation period.

#### Immobilization of protease

An aqueous agar solution 5% (w/w) was prepared by heating it at the boil. The agar solution was cooled down and left to solidify in a Petri-dish. A cork borer was used to cut the obtained agar gel into disks. The agar disks were activated after impregnating in a sericin–polyethylene–imine (PEI) solution of definite concentrations and pH for 2 h. The activated disks were meticulously washed and then soaked in a glutaraldehyde (GA) solution (5%, v/v) for 1 h. The obtained agar disks were kept in distilled water at low temperature till they were loaded with protease enzyme. In order to attain the highest amount of immobilized protease, the following three factors were optimized using the Box–Behnken design (BBD): sericin concentration (A), the PEI concentration (B), and the sericin-PEI solution pH (C). Each factor was investigated at three levels throughout 17 experimental runs Table 1. The attained immobilized protease activities were given as U g^−1^. The activity recovery percent (AR%) was also calculated given that I is the immobilized protease activity, and L is the activity of the loading protease solution.

$${\text{AR}}\% \, = {\text{ I}}/{\text{L}} \times {1}00$$

**Table 1:** Box–Behnken design (BBD)

Run	A: Sericin conc. (%, w/w)	B: PEI conc. (%, w/w)	C: Sericin-PEI (pH)	Protease activity (U g^−1^)
1	0 (2)	0 (4)	0 (8)	376.10
2	0 (2)	0 (4)	0 (8)	370.80
3	0 (2)	0 (4)	0 (8)	380.43
4	1 (4)	0 (4)	1 (9)	353.13
5	− 1 (0)	− 1 (1)	0 (8)	159.30
6	1 (4)	− 1 (1)	0 (8)	244.70
7	0 (2)	− 1 (1)	− 1 (7)	179.10
8	0 (2)	− 1 (1)	1 (9)	154.47
9	0 (2)	1 (7)	− 1 (7)	201.80
10	1 (4)	0 (4)	− 1 (7)	230.73
11	1 (4)	1 (7)	0 (8)	283.13
12	− 1 (0)	0 (4)	− 1 (7)	111.90
13	0 (2)	0 (4)	0 (8)	318.53
14	− 1 (0)	0 (4)	1 (9)	188.90
15	− 1 (0)	1 (7)	0 (8)	170.97
16	0 (2)	1 (7)	1 (9)	216.83
17	0 (2)	0 (4)	0 (8)	463.17

#### Evaluation of protease catalytic activity

##### pH stability

Enzyme activity of both free and immobilized enzyme was measured at 55 °C at different pH values using (citrate buffer pH from 4.0 to 5.0, 0.2 M phosphate-buffer pH from 6.0 to 8.0 and glycine–NaOH buffer pH 9 and10). The pH stability within an acidic to basic region (4–10) was assigned. The enzyme solution was incubated with the tested buffer for different duration times (30 and 60 min) at 30 °C and the residual activity was assessed.

#### Optimum temperature and thermal stability

The optimum temperature for the isolated enzyme (free and immobilized) was monitored by incubation of the reaction mixture of the enzymes at a temperature range of 30–70 °C. The thermal stability of the isolated thermophilic protease was investigated by its incubation at 30–80 °C for different incubation periods, 15, 30 and 45 min in the absence of substrate, and the relative activity was determined for both the free and immobilized thermophilic protease.

#### Kinetics studies of free and immobilized protease

The Lineweaver–Burk plot (double reciprocal) method (Lineweaver and Burk 1934) was adopted to obtain the Michaelis–Menten kinetic models, adequate for the description of the hydrolysis of casein by the isolated free and the immobilized thermophilic protease. Apparent *K*_*m*_ and *V*_max_ of free and immobilized protease were determined by plotting 1/[*S*] against 1/[*V*], respectively, where [S] is the casein (substrate) concentration, *V*_*o*_ is the initial enzyme velocity, *V*_max_ is the maximum enzyme velocity, and *K*_*m*_ is the Michaelis constant and is defined only in experimental terms and equals the value of [S] at which *V*_*o*_ equals ½*V*_max_. A substrate concentration was in the range of 1–7 mg/mL at 60 and 65 °C for free and immobilized enzyme respectively, and pH8.0 for 30 min.

#### Bio-treatment of wool

Wool fibres were bio-treated with various amounts (5–25%, v/v) of the extracted thermophilic protease at a temperature range from 35 and 60 °C; the treatment time was 1–24 h, the pH was 5–9, and the liquor ratio was 1:50. The bio-treated fibres were thoroughly rinsed with tap water and left to dry at ambient temperature.

In another trial, wool fibers were bio-treated with the immobilized form of the said enzyme using 25 loaded discs (ca. 1150 unit) with TP enzyme for different periods of time, pH 7, 55 °C and liquor ratio: 1:50. The reusability of the immobilized enzyme was investigated by bio-treatment of wool with the immobilized enzyme for up to six times, 24 h each.

#### Dyeing

The affinity of bio-treated wool fibres towards the anionic dye C.I. Acid Blue 203 was compared to that of the corresponding untreated one. The treated and untreated samples were dyed in an aqueous solution (1% shade) of the said dye for 1 h at 90 °C and pH 4.5 (adjusted using acetic acid); the liquor ratio was 1:50. The dyed wool samples were rinsed with running water, followed by air-drying at room temperature.

#### One-bath consecutive bio-treatment and dyeing

Aiming to save energy and water that are extensively consumed during dyeing and finishing of textiles, wool tops were treated with 25% (v/v) TP enzyme at 55 °C for 8 h at pH 7; the liquor ratio was 1:50. The bio-treated wool sample was removed from the bath to prepare it for the consecutive dyeing operation using C.I. Acid Blue 203 (1% shade). The temperature was raised to 90 °C and the pH was adjusted at 4.0 (using acetic acid). The bio-treated sample was then returned to the bath and the dyeing operation was continued for 1 h with occasional gentle shaking (60 rpm).

### Analyses and testing

#### Dye exhaustion

The dye absorbance was measured at “0” dyeing time (*A*_*i*_) and at the end of the dyeing process (*A*_*f*_). The measurement was carried out at *λ*_max_ 575 nm on a JENWAY-6405 UV/Vis spectrophotometer (Bibby Scientific Ltd, UK). The percent of dye exhaustion (E %) was calculated from the measured absorbance using the following relation:

$${\text{E}}\% = \left[ {\left( {A_i -A_f } \right)/A_i } \right] \times 100$$

#### Weight loss

The fabric weight loss due to bio-treatment of wool tops was calculated according to the following equation:

$${\text{Weight loss}}\left( \% \right) = \, [(W_1 - \, W_2 )/W_1 ] \times 100,$$

where *W*_1_ and *W*_2_ are the weights of fabric before and after the enzyme treatment.

#### Determination of felting shrinkage of wool

The felting of wool fibres was determined according to the Aachener felting test using three-dimensional shaking machine according to the standard test method IWTO 20-2004.

#### Amino acid analysis

Wool samples were hydrolysed using 6 N hydrochloric acid at 105 °C for 24 h. The amino acid composition of the hydrolysate was determined using “Alpha Plus II” Amino Acid Analyzer.

#### Water analysis

Characterization of the residual treatment bath was brought about by determining its chemical oxygen demand (COD) according to 5220-COD standard test method, biological oxygen demand (BOD) according to 5210-BOD standard test method, total suspended solid (TSS), and total dissolved salt (TDS).

#### Alkali solubility

The alkali solubility of the bio-treated as well as untreated wool is taken as a measure of the number of disulphide crosslinks along keratin macromolecules. It was assessed in accordance with the International Wool Textile Organization (IWTO)-4-66(D) test method.

#### Whiteness index

The degree of whiteness of wool samples was evaluated using a Macbeth Colour Eye MS 2020 spectrophotometer.

#### Fibre morphology

The surface morphology of the protease treated and untreated wool fibres were examined by using Bruker Nano GmbH Scanning Electron Microscope D-12489 Berlin, Germany. The samples were mounted on aluminium stubs, and sputter coated with gold in an S150A with 20 kV scanning voltage.

#### Determination of the bundle strength and elongation

The fibre strength and elongation of wool fleece was assessed by adopting the standard method IWTO-32-82(E) using a tensile tester machine (Instron).

## Results and discussion

### Screening and isolation of protease producing bacteria

**Fig. 1:**
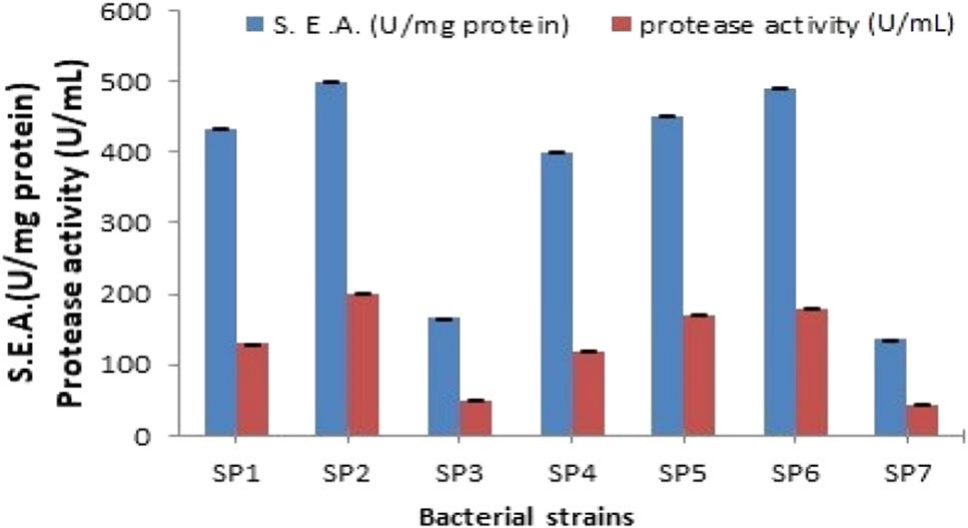
Specific enzyme activities (S.E.A.) and protease activities estimated for the different bacterial isolates (SP1 to SP7) in order to select the most potent protease producing isolate

To find out the efficiency of bacterial cultures in producing proteolytic enzymes, seven isolated strains were tested. The maximum protease activity and specific activity (200 ± 1 U/mL and 500 ± 0.57 U/mg protein) were obtained by the bacterial strains SP2 (Fig. 1). The promising isolated strain was identified using polymerase chain reaction 16S rDNA and electrophoresis analysis and registered in Genbank.

### Identification and phylogenetic analysis (SP2)

Strain SP2 identification was determined by the 16S rRNA gene sequence (1000 bp). The results indicated that strain SP2 16S rRNA gene sequence was similar to that of many species of the genus *Bacillus* according to the GenBank database and a BLAST search. These results showed that the isolate SP2 is closely related to the *Bacillus safensis* FO-36b (GenBank Accession no. MZ836779) with 97.76% identity.

### Effect of incubation periods on protease production

**Fig. 2:**
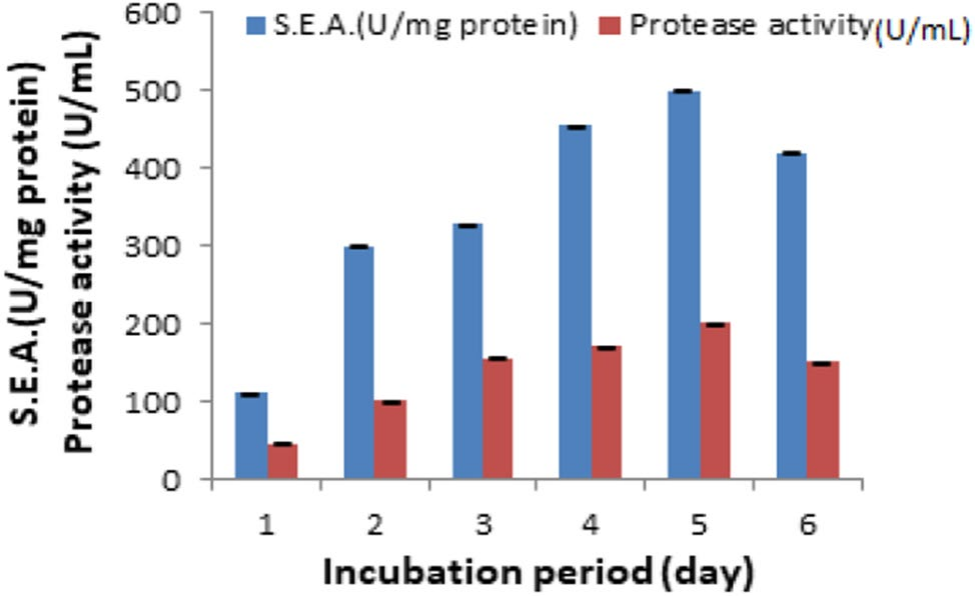
Effect of altering the incubation periods on the protease activity and specific activity (S.E.A.) offered by *B. safensis* FO-36bMZ836779

The time required for the optimum protease production by bacterial strain was determined. The results in Fig. 2 indicate that the maximum production of protease (200 ± 0.58 U/mL and 500 ± 1 S.E.A U/mg protein) by *B. safensis* FO-36bMZ836779 was obtained after 5 days’ incubation by the shaken cultures in mineral medium containing wool as sole carbon and nitrogen source. Similar result was obtained by Hassan et al. (2013) who found that alkaline keratinolytic serine protease produced after 5 days by *Bacillus amyloliquefaciens* MA20 and *B. subtilis* MA21. It was reported that the optimum incubation time for protease production by *Bacillus licheniformis* and *Bacillus coagulans* is was 96 h (Asokan and Jayanthi 2010).

### Immobilization of protease enzyme

#### Optimization of the activated agar carrier via BBD

1 $$\begin{aligned} {\text{Amount of immobilized protease}} & = { 381}.{81} + {6}0.08A \\ & \,\,\,\, + 16.90B + 23.72C + 6.69AB \\ & \,\,\,\, + 11.35AC + 9.92BC - 67.08A^2 \\ & \,\,\,\, - 100.20B^2 - {93}.{56}C^{2} \\ \end{aligned}$$

A quadratic model was utilized to analyse the results attained by the BBD Table 1. This model possessed a 0.89 *R*^2^, and this confirmed its validity as it could account for 89% of the alterations among the results. Table 2 revealed that the model presented a *P* value of 0.0107 which indicated its significance. The model equation, which related the amount of immobilized protease to the coded values of the three tested factors, was presented as Eq. (1). The optimal activation protocol proposed after the results’ analysis comprised soaking the agar disks in a 4.3%PEI-2.9% sericin solution of pH 8.2. This optimal treatment would allow for the attainment of 398.6 U g^−1^ immobilized protease. Taking into consideration that 1 g of the activated agar carrier was loaded with 2000 U of protease, it could be seen that a 19.93% activity recovery would be attained via the optimally activated agar carrier. This percent was larger than the 13.12% activity recovered via the PEI-GA activated agar carrier, which was not co-processed with sericin, after immobilizing β-galactosidase (Wahba and Hassan 2015).

**Table 2:** Analysis of variance (ANOVA)

Source	SS^a^	DF^b^	Mean	*F* value	*P* value
Model	145,930.21	9.00	16,214.47	6.56	0.0107
A-Sericin concentration	28,875.65	1.00	28,875.65	11.68	0.0112
B-PEI concentration	2283.75	1.00	2283.75	0.92	0.3685
C-Sericin-PEI pH	4502.85	1.00	4502.85	1.82	0.2192
*AB*	179.07	1.00	179.07	0.07	0.7956
*AC*	515.29	1.00	515.29	0.21	0.6618
*BC*	393.30	1.00	393.30	0.16	0.7019
*A*^2^	18,948.10	1.00	18,948.10	7.66	0.0278
*B*^2^	42,273.15	1.00	42,273.15	17.10	0.0044
*C*^2^	36,854.10	1.00	36,854.10	14.91	0.0062
Residual	17,305.61	7.00	2472.23		
Lack of fit	6527.04	3.00	2175.68	0.81	0.5520
Pure error	10,778.56	4.00	2694.64		
Cor total	163,235.82	16.00			

^a^Sum of squares

^b^Degrees of freedom

**Fig. 3:**
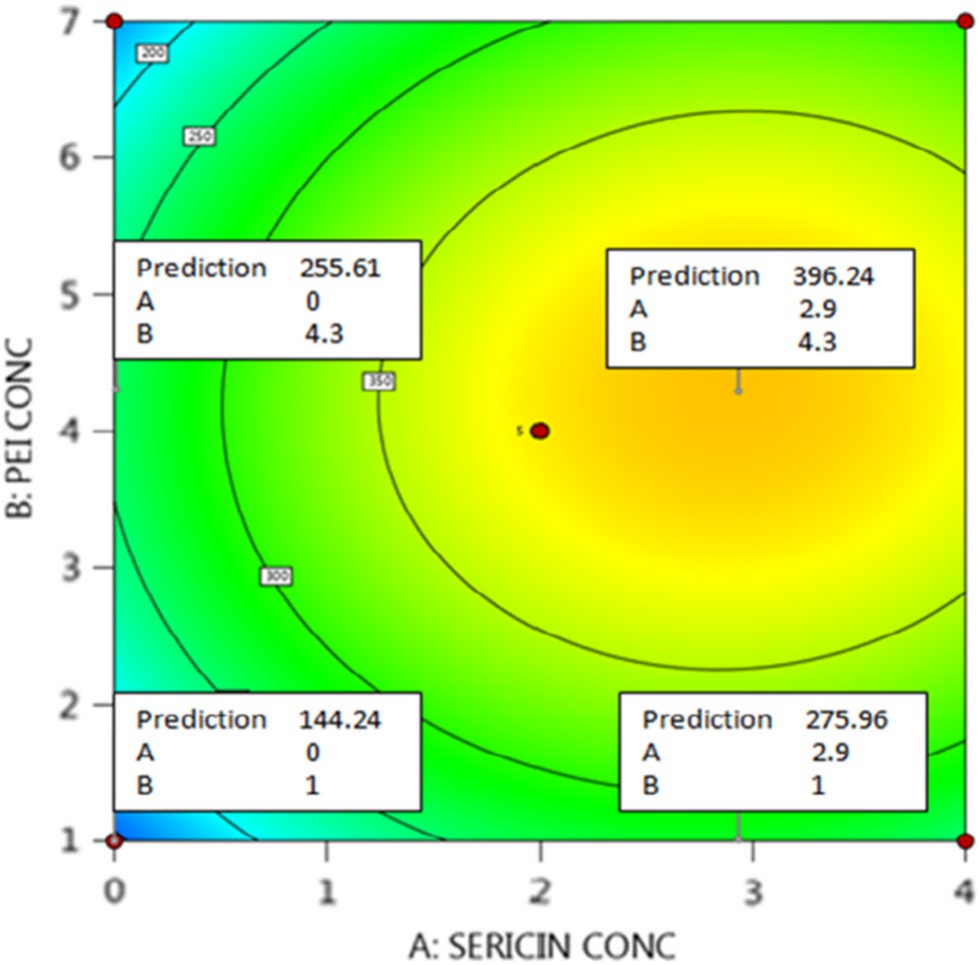
Contour plot which explored the effects of altering both the sericin concentration (*A*) and the PEI concentration (*B*) on the amount of immobilized protease [pH was constant at 8 (0 level)]

Table 2 reveals that the quadratic terms of the three tested factors were significant. Moreover, the linear term for the sericin concentration (*A*) also significantly affected the amount of immobilized protease where it presented a *P* value of 0.0112. The significance of supplementing the activating PEI solution with sericin could be further clarified from Fig. 3 which explored the effects of altering both the sericin concentration (*A*) and the PEI concentration (*B*) whilst retaining their pH constant at 8 (0 level). Figure 3 predicts that the amount of immobilized protease would increase from 144.24 to 275.96 U g^−1^ if a 1% PEI solution was supplemented with 2.9% sericin. Furthermore, an increase from 255.61 to 396.24 U g^−1^ would be attained if a 4.3% PEI solution was supplemented with 2.9% sericin. PEI is commonly utilized to activate biopolymers and enable them to covalently immobilize enzymes owing to its poly-amine nature. The PEI cationic amine moieties cross-link the anionic moieties of biopolymers, such as the sulfate and the pyruvate moieties of agar, and this links the PEI to the biopolymer. Moreover, the PEI nucleophilic amine moieties bind to GA, which is the functional moiety responsible for the covalent immobilization of enzymes (Wahba and Hassan 2017). The poly-amine nature of other compounds, such as the polysaccharide chitosan (Wahba 2017) and the proteinaceous whey protein isolate (Wahba and Soliman 2018), also enabled these compounds to successfully activate biopolymers and provide the necessary amine moieties for the ionic cross-linking with the biopolymers and the covalent binding to GA. Sericin also exhibits a polyamide nature as it is a protein (Yang et al. 2015). Thus, it might also be utilized to activate biopolymers. Nevertheless, at the tested pH range (7–9) sericin would not probably provide the cationic amine moieties required to ionically cross-link the anionic agar as sericin possess an isoelectric point of 4.3 (Wang et al. 2014) and it formerly was shown to be predominantly anionic at pH 6–7 (Yang et al. 2015). Thus, it did not in itself bind to the anionic agar. Sericin could have bound to the agar-PEI complex through ionically cross-linking the cationic PEI moieties via its anionic moieties which would be prevalent at the tested pH range (Yang et al. 2015). Afterwards, sericin could have provided additional nucleophilic amine moieties to interact with GA. This could have caused more GA to bind to the PEI-sericin activated agar disks and could have caused the amount of immobilized protease to increase after adding sericin.

### Evaluation of protease catalytic activity

#### Effect of pH

**Fig. 4:**
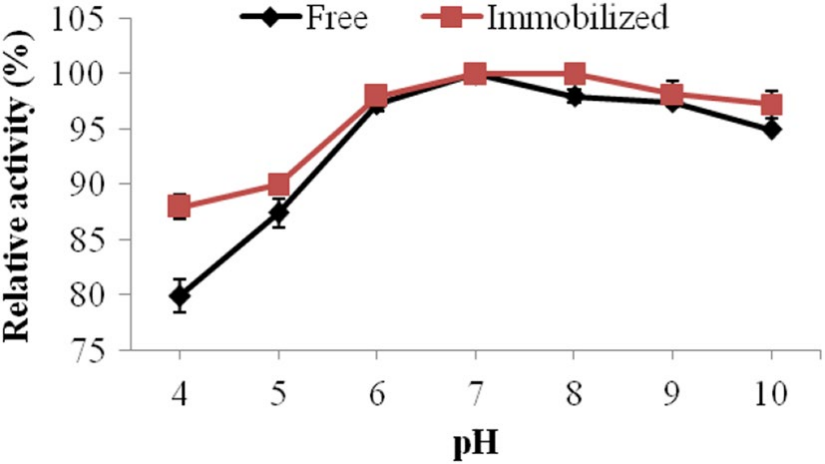
Activities offered by the free and immobilized proteases at different pH values

The main goal of this work was to determine the optimal conditions of pH at which the protease enzyme can be applied in the textile sector. In Fig. 4 it can be seen that optimal pH of free enzyme was 7.0 and that of immobilized enzyme was between 7.0 and 8.0. Noteworthy, an alkaline shift also occurred in cholesterol oxidase optimal pH secondary to its immobilization (Huang et al. 2015). The variations observed in immobilized enzymes pH optima could be regarded to the conformation alterations suffered by the enzymes after their covalent immobilization. Moreover, the immobilization carrier would alter the micro-environment of the enzyme, and this might also cause such pH variations (Wahba 2022).

**Fig. 5:**
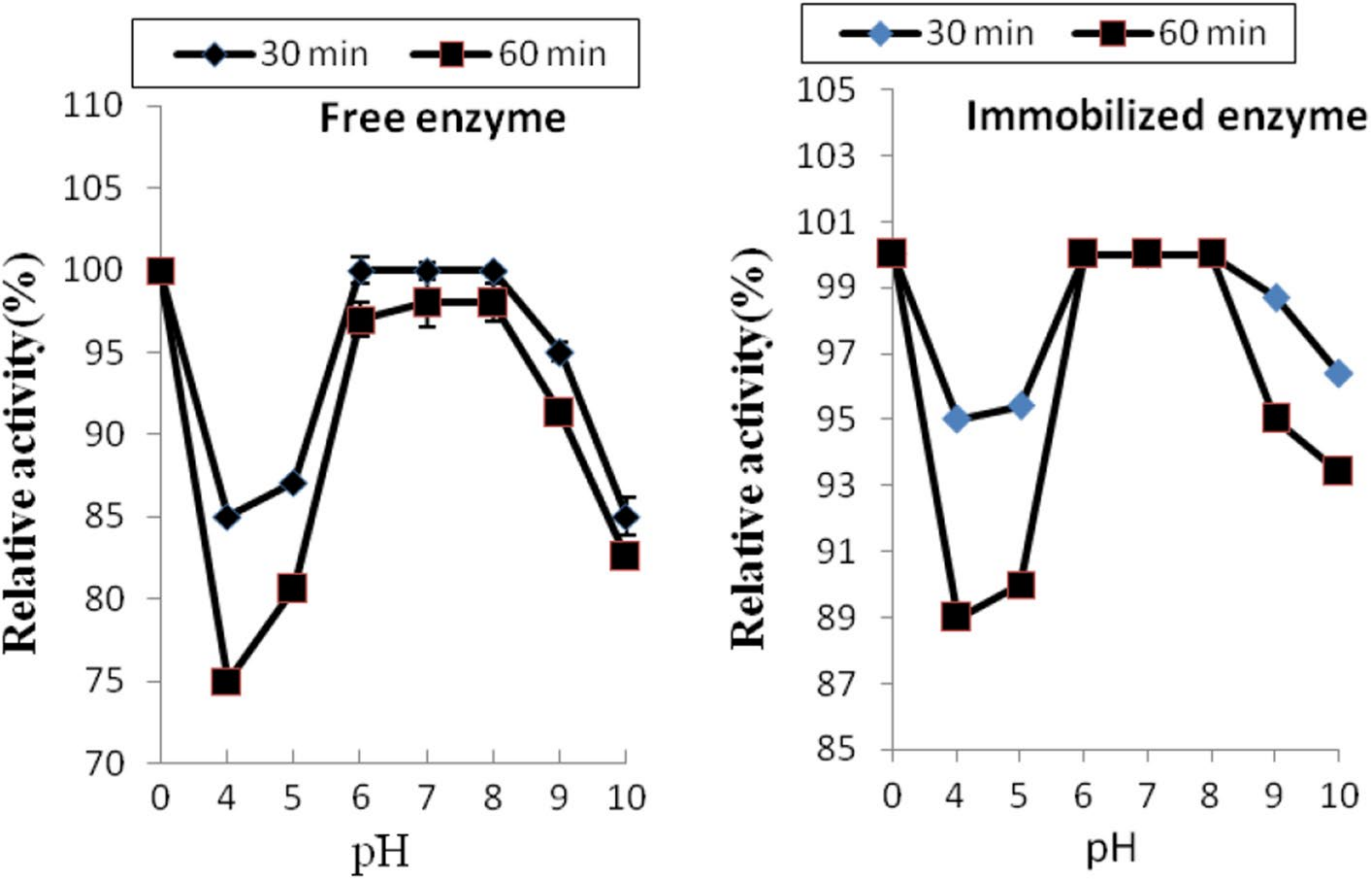
Effect of altering pH on the stability of free and immobilized proteases where the enzymes were incubated with the tested buffers for either 30 or 60 min at 30 °C before estimating their residual activities

The stability of the free and immobilized protease at various pHs (4–10) for 30 and 60 min was presented in Fig. 5. It was revealed that the immobilized enzyme was more stable for a longer time than the free enzyme; hence, it could be used for the industrial application.

#### Effect of temperature

**Fig. 6:**
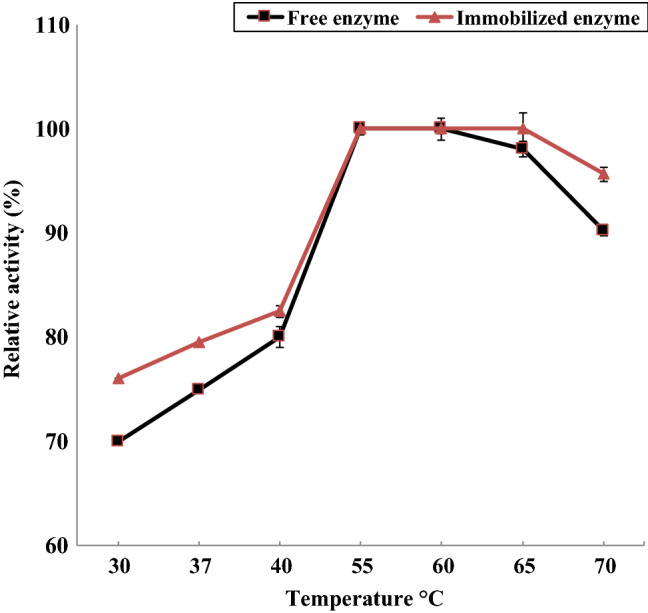
Activities offered by the free and immobilized proteases at different temperatures

**Fig. 7:**
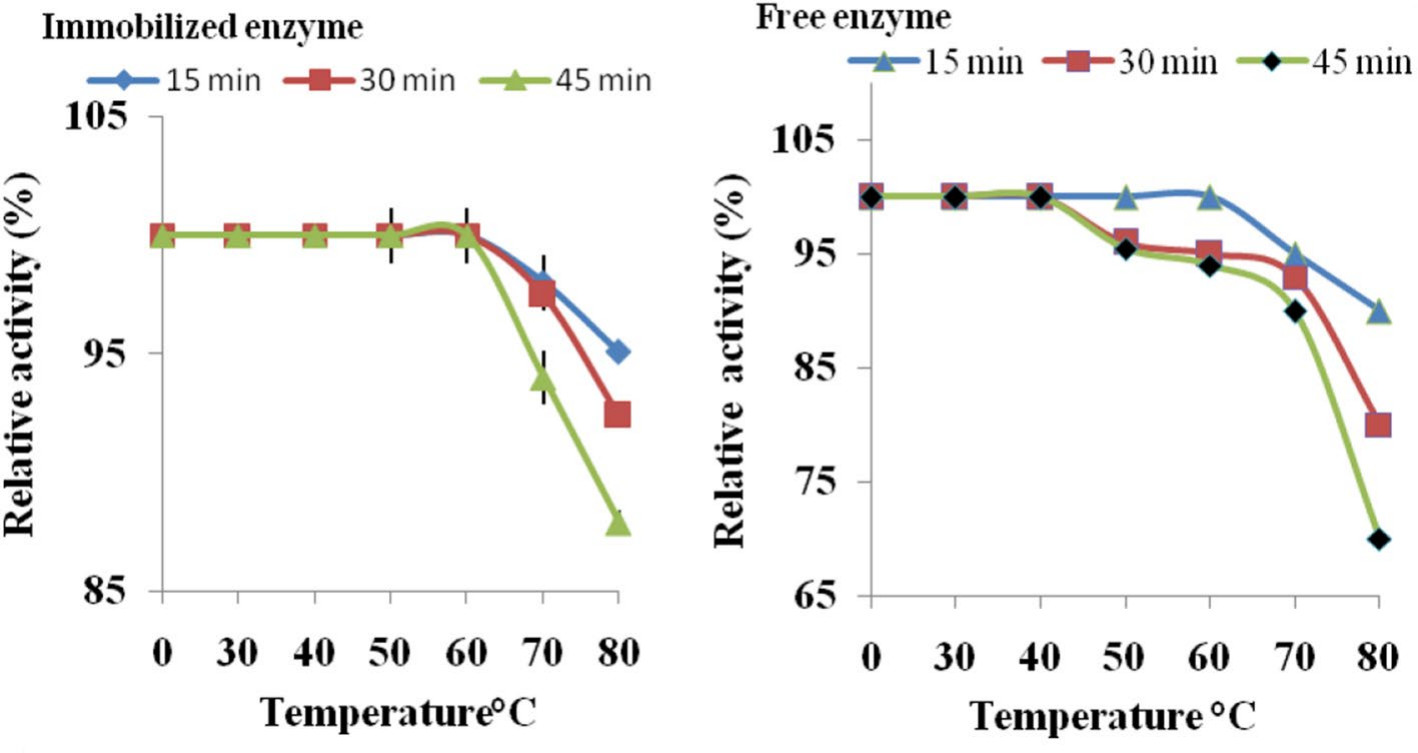
Thermal stability of free and immobilized proteases where the enzymes were incubated at 30–80 °C for 15, 30 and 45 min in the absence of substrate. Afterwards, their relative activities were determined

In Fig. 6, the optimum temperature of the produced free and immobilized protease was at 55 and 60 °C, respectively. This was in agreement with previous reported investigations (Manni et al. 2020). The immobilized enzyme also exhibited its maximum 100% activity at temperature 65 °C whereas the free enzyme offered 98.1 ± 0.6% activity at the same temperature. On increasing the temperature to 70 °C, a minimal decline in immobilized enzyme activity occurred and 95.7 ± 0.5% activity was recorded. The increments in optimum temperatures after immobilization might be regarded to the establishment of covalent linkages betwixt the carrier and the enzyme. These linkages would rigidify the enzyme configuration and would cause it to be less influenced by temperature denaturation (Awad et al. 2020). In Fig. 7, the immobilized protease remained 100% active at temperatures up to 60 °C for 45 min, whereas the free protease was 100% active at 50 and 60 °C for 15 min. The effect of higher temperatures (70–80 °C) on the enzyme activity was more pronounced in case of the free enzyme than the immobilized one. Noteworthy, immobilization could also restrict the interactions betwixt enzyme entities, and this might help preserve the enzymes activity at incremented temperatures (Awad et al. 2020), especially in case of proteases which could be subjected to autolysis. That is immobilization of proteases could reduce autolysis (Sharma et al. 2003) and thermal denaturation (Ferreira et al. 2003; He et al. 2000), and this is favorable in industries (Elnashar and Hassan 2014).

### Kinetics studies

*K*_*m*_ for free and immobilized protease was calculated using the double reciprocal plot method Lineweaver–Burk plot in Figure S1. *K*_*m*_ and *V*_max_ values (1.88 mg/mL and 181.8 U/mg protein, respectively, for free enzyme and 2 mg/mL and 192.3 U/mg protein, respectively, for immobilized enzyme). The increase in *K*_*m*_ implied that the substrate affinity of the immobilized enzyme was diminished. This diminished affinity could have been caused by the limitations imposed on the substrates diffusion. Furthermore, immobilization would rigidify the enzyme configuration, and rigidified enzymes might be less capable of creating the enzyme–substrate complex. Thus, their substrate affinity would be diminished (Wahba 2018).

### Bio-treatment of wool with free protease

The production of machine-washable woolen garments is a customer-oriented demand, and the application of green technology to attain this goal is an eco-driven task (Allam et al. 2009). Herein, wool tops were treated with a thermophilic protease (TP) (*B. safensis* FO-36bMZ836779) by adopting different reaction conditions; namely enzyme concentration, treatment time, treatment temperature, and pH. Results of this investigation are abridged in Tables 3, 4, 5 and Table S1.

**Table 3:** Effect of treatment temperature on weight loss and the felting resistance of wool fibre using *B. safensis* FO-36bMZ836779 [Bio-treatment conditions: 50%v/v (10,000 unit) TP enzyme, pH 7, 24 h, and liquor ratio 1:50]

Temperature (°C)	Loss in weight (%)	Felt ball diameter (cm)
Untreated	–	2.12
35 °C	9.1	2.85
40 °C	9.5	Deformed ball
45 °C	15.45	No ball
50 °C	18.6	No ball
55 °C	24.2	No ball
60 °C	9.3	2.88

**Table 4:** Effect of treatment time on weight loss and the felting resistance of wool fibre using *B. safensis* FO-36bMZ836779 [Bio-treatment conditions: 25% TP protease (5000 unit), 55 °C, pH 7, and liquor ratio 1: 50]

Time (h)	Loss in weight (%)	Felt ball diameter (cm)
1.0	2.77	2.08
1.5	3.37	2.13
3.0	3.04	2.32
6.0	3.3	2.47
8.0	5.63	Deformed ball
10.0	9.8	Deformed ball
12.0	16.6	No ball
24.0	20.7	No ball

**Table 5:** Effect of pH on the felting resistance of wool fibre treated with *B. safensis* FO-36bMZ836779 [Bio-treatment conditions: 25% (5000 unit) TP enzyme at 55 °C, 8 h, and liquor ratio: 1:50]

pH	Loss in weight (%)	Felt ball diameter (cm)
Untreated	–	2.12
5	2.62	2.13
6	6.33	Deformed ball
7	5.63	Deformed ball
8	3.69	2.72
9	1.25	2.17

Table 3 shows the effect of bio-treatment temperature on the weight loss and felt ball diameter of wool fibres subjected to shaking in three-dimensional shaking machine. Data of this table implies that bio-treatment of wool tops with TP enzymes at 35 °C resulted in remarkable improvement in their felting resistance, as indicated by increasing the felt ball diameter from 2.12 cm, for untreated wool, to 2.85 cm in case of enzyme-treated wool. Machine washable wool was obtained upon treatment of wool tops with TP enzyme at 40, 45, 50, and 55 °C as according to the International Wool Textile Organization (IWTO) standard test method, “No ball” and “Deformed ball” indicate the highest resistance of wool to felting (IWTO 2017). The fascinating felt-proofing effect on wool fibres of the used enzyme is due to its ability for partial descaling of wool fibre surface, a step which is mandatory to obtain machine-washable wool in such treatments (El-Sayed et al. 2010b). Further increase in treatment temperature to 60 °C still produced wool fibres with improved felting resistance, but not to the extent of machine-washable grades. This reflected the fact that although the used protease is thermophilic, yet it withstood such temperature not for a long time (c.f. Figs. 6, 7). The loss in weight of the bio-treated wool tops ranged between 9.1 and 24.2% depending on the treatment temperature, which is beyond the acceptable limit.

The effect of TP concentration on the felting resistance is shown in Table S1. It is clear from this table that 25% (v/v) of the used free TP enzyme is adequate to render machine-washable wool (as indicated by no ball formation after the felting test). The loss in weight (20.7%) is still far beyond the acceptable limits.

Table 4 shows the effect of bio-treatment time on the weight loss and felting resistance of wool fibres. It is clear from data that treatment of wool with the used TP enzyme for 8 h resulted in wool fibres with superior resistance to felting shrinkage without severe fibre deterioration (the loss in weight is 5.63%). Further increase in the treatment time led to severe deterioration to the fibre. We conclude that the TP enzyme digests wool tops in a layer-wise way. Within the first 8 h, the enzyme digests the outermost cuticle layer of wool resulting in felt-proofed wool without affecting the fibre interior. Prolonged treatment time resulted in extending the digestive action of the enzyme towards the subsequent cuticle layers and may harm the fibre cortex.

The effect of the pH of the treatment bath of wool with TP enzyme on its felting shrinkage was investigated and the results are summarized in Table 5. Data of this table indicate that only at pH 6 and 7 the said enzyme can be utilized in treatment of wool to enhance its resistance to felting shrinkage to the extent of obtaining machine-washable fabrics. This is in harmony with the results of the activity measurements of the used TP enzyme (c.f. Figs. 4, 5).

### Bio-treatment of wool with the immobilized protease

In an attempt to increase the thermal stability and reusability of the used TP protease, the free enzyme was immobilized onto sericin-PEI-GA agar carrier. Table 6 shows the effect of treatment of wool with the immobilized TP (ITP) protease enzyme on its felting resistance. Data in this table imply that, at the same reaction conditions, the effect of the used ITP enzyme on wool tops is less than that of its analogous free enzyme. For instance, the measured diameter of the felt ball of wool sample subjected to the Aachener felting test was 2.55 cm for wool tops treated with the ITP enzyme while the corresponding sample treated with the free enzyme resulted in a deformed ball. Machine-washable wool can be obtained only upon treatment of wool tops with the ITP protease for 24 h. Data of this table imply also that the ITP protease can be used for up to five times in felt-proofing of wool. This result assures that the use of ITP protease in manufacture of super wash wool tops would have positive impact on the economic cost of the wet processing of wool.

**Table 6:** Felting resistance of wool fibres bio-treated with Immobilized *B. safensis* FO-36bMZ836779 [Bio-treatment conditions: 25 disc (ca. 1150 unit), pH 7, 55 °C, and liquor ratio: 1:50]

Treatment of wool with	Loss in weight (%)	Felt ball diameter (cm)
ITP enzyme for 8 h (first time)	3.9	2.55
ITP enzyme for 16 h (first time)	4.6	2.86
ITP enzyme for 24 h (first time)	6.4	Deformed ball
ITP enzyme for 24 h (second time)	6.5	Deformed ball
ITP enzyme for 24 h (third time)	6.3	Deformed ball
ITP enzyme for 24 h (fourth time)	5.7	3.24
ITP enzyme for 24 h (fifth time)	5.1	3.12
ITP enzyme for 24 h (sixth time)	4.9	2.61

### Dyeing of wool

Enzyme-treated wool was dyed with C.I. Acid Blue 203 either in a new bath or as two consecutive processes in the same bath. The latter procedure was adopted to reduce fresh water and energy consumption during dyeing and finishing of wool. One-bath consecutive bio-treatment and dyeing was carried out by removing the bio-treated sample from the bath, and temperature raised from 55 to 90 °C and the pH was adjusted to 4, and finally the sample was returned to the bath.

Results of this study, summarized in Table 7, clarify that consecutive bio-treatment and dyeing of wool in the same bath resulted in similar dye exhaustion to those untreated and dyed, or bio-treated then dyed in a new bath. The use of hot water produced from bio-treatment of wool would save energy and water; the former is the most expensive component in textile sector.

**Table 7:** Dyeability of untreated as well as bio-treated wool tops towards C.I. Acid Blue 203 (Dyeing conditions: 1% dye shade, 90 °C, pH 4, and liquor ratio: 1: 50)

Sample	Dye exhaustion (%)
Untreated wool	95.6
Bio-treated wool with free TP protease followed by dyeing	96.6
Bio-treated wool with free TP protease followed by dyeing in the same bath^a,b^	94.1
Bio-treated wool with the ITP protease followed by dyeing	90.2

^a^Bio-treatment conditions: 25% (v/v) TP protease, at 55 °C, pH 7, for 8 h; liquor ratio: 1:50

^b^The bio-treated sample was removed from the bath, the temperature and pH were adjusted for the dyeing process, and the bio-treated sample was returned to the bath

### Fibre morphology

**Fig. 8:**
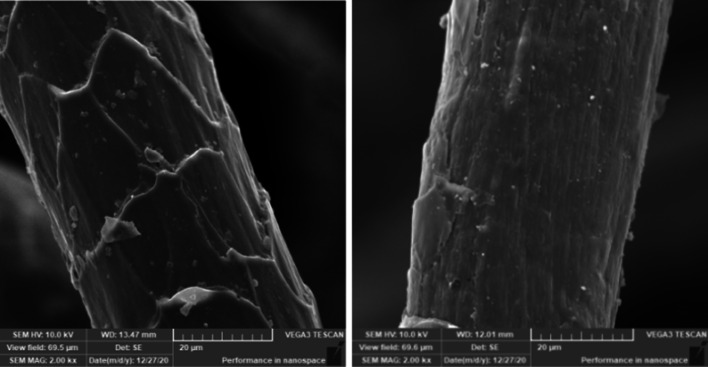
Scanning electron micrographs of untreated (left), and bio-treated (right) wool tops [Bio-treatment conditions: 25% (5000 unit) TP enzyme at 55 °C, 8 h, and liquor ratio: 1:50]

The surface morphology of the bio-treated as well as untreated wool tops was examined using scanning electron microscope. Figure 8 reveals that the scaly structure of wool fibre surface was partially removed without deterioration upon bio-treatment with TP protease. This is one of the essential subtractive procedures which are usually followed to obtain machine-washable woolen goods with minimum loss in weight and limited effect on the inherent properties of wool (El-Sayed et al. 2002).

### Water analysis

One of the main advantages of bio-treatment of textiles is the limited pollutants which might be discharged into the effluent (Abou Taleb et al. 2022). Therefore, after bio-treatment of wool with the extracted TP protease, the discharged water was characterized by testing its chemical oxygen demand (COD), biological oxygen demand (BOD), total dissolved salt (TDS), and total suspended salt (TSS). The results of this investigation, shown in Table 8, imply that the pH, COD, BOD, and TSS values of the discharged effluent from bio-treatment of wool with TP protease are within the permissible limits according to the Egyptian legislations [Ministerial Decree (44/2000)] (El-Sayed et al. 2019). This constitutes one of the brightest advantages of the proposed method in comparison with the polluting commercially used felt-proofing of wool (El-Sayed et al. 2022).

**Table 8:** The characteristics of water discharged from bio-treatment of wool using *B. safensis* FO-36bMZ836779

Parameter	Tap water/effluent from bio-treatment of wool	Ministerial Decree (44/2000)
pH	7.2/6.7	6–9.5
TSS (mg/L)	0/414	800
COD (mgO_2_/L)	10/1082	1100
BOD (mgO_2_/L)	3/532	600
TDS (mg/L)	15/791	–

### Amino acid analyses of wool

The alteration in the percent of each amino acid in wool keratin macro-molecules after being bio-treated with TP protease was assessed and the results thereof are summarized in Table S2. Data of this table depict that there is a remarkable increase in some amino acid contents in the bio-treated wool. These include phenyl alanine (45.3%), proline (45.1%), threonine (25.9%), alanine (14.1%), and glutamic acid (12.1%). The contents of other amino acids were lessened to large extents: Viz. cystine (39.6%), aspartic acid (34.9%), methionine (22.1%), lysine (21.4%), and serine (11.1%). Valine and leucine are the least affected amino acids.

These findings elucidate that the specificity of the used TP protease is limited and can attack peptide bonds at different amino acid residues along wool keratin macromolecule. Hence, there is adverse decrease in the amounts of various types of amino acids: Viz. acidic amino acid (aspartic acid), basic amino acid (lysine) and sulphur-containing amino acids (cystine and methionine). The effect of the used TP on the amino acid composition of wool is similar, in most cases, to that of dilute solutions of alkalis (Vineis et al. 2021).

#### Characterization of wool

The effect of treatment of wool with the extracted TP protease on some of its inherent properties was investigated and the results tabulated in Table 9. It was seen the alkali solubility and the tenacity of enzyme-treated wool tops decreased by ca. 19.5 and 10.3%, respectively, relative to the untreated sample. This may be attributed to the rupture of some of the disulphide bonds between keratin macromolecules which weakens the fibres and makes them more susceptible to the effect of alkalis (Abou Taleb et al. 2020b).

**Table 9:** Effect of bio-treatment of wool with TP protease on some of its inherent properties

Wool property	Untreated wool	Bio-treated wool
Alkali solubility (%)	13.0	15.46
Whiteness Index	− 21.48	− 2.75
Tenacity (cN/tex)	5.11	4.58
Elongation at break (%)	9.142	9.918

On the other hand, the degree of whiteness of wool increases sharply under the influence of the used enzyme. Moreover, the elongation at break of the bio-treated fibres was enhanced to a remarkable extent. It has been reported that partial removal of the cuticle scales on the surface of wool fibres has a positive impact on their degree of whiteness (Ammayappan 2013).

## Conclusion

A new thermozyme was isolated from hot region in Egypt. The phylogenetic study showed that the isolate is closely related to the *B. safensis* FO-36b with 97.76% identity (GenBank Accession no. MZ836779). Sericin was found to be a significant constituent of the protease immobilizer which enhanced the stability of the enzyme.

We concluded that the extracted thermozyme is a suitable reagent for production of machine-washable wool without severe deterioration of the fibres’ inherent properties. Furthermore, the said enzyme can be used in a one bath/two successive steps process for anti-felting and dyeing of wool tops with anionic dye. Therefore, the use of TP protease for wool processing can result in reducing energy and water consumption.

The scanning electron micrograph of the bio-treated wool emphasized that treatment of wool with the extracted TP partially removed the scales on the fibre surface without aggressive effect on the bulk of the fibres. The amino acid analysis of the bio-treated wool showed that the used enzyme is not specific to attack polypeptide chains at specific amino acid residues. Analysis of the drained water from the bio-treatment process emphasizes that the COD, BOD, TSS, and TDS are within the permissible limits according to the environmental laws.

## Supplementary Information

Below is the link to the electronic supplementary material.

Supplementary file1 (DOC 95 KB)

## Data Availability

The datasets used and analysed during the current study are available from the corresponding author on reasonable request.
