# Supplementary material for: Green method for improving performance attributes of wool fibres using immobilized proteolytic thermozyme
Source: 3 Biotech. 2022 Sep 2;12(10):254. doi: 10.1007/s13205-022-03323-y (PMC9440185; doi:10.1007/s13205-022-03323-y)
Supplement: Supplementary file 1 — Supplementary file1 (DOC 95 KB) [file 13205_2022_3323_MOESM1_ESM.doc]

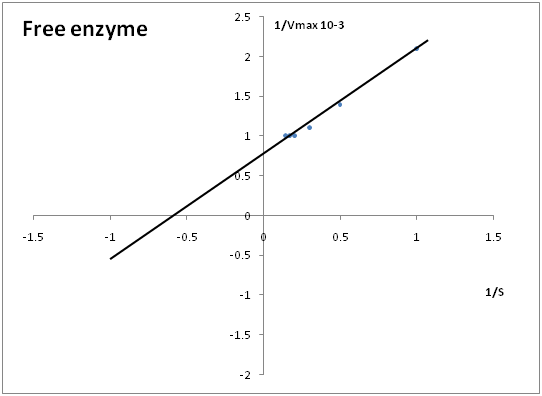

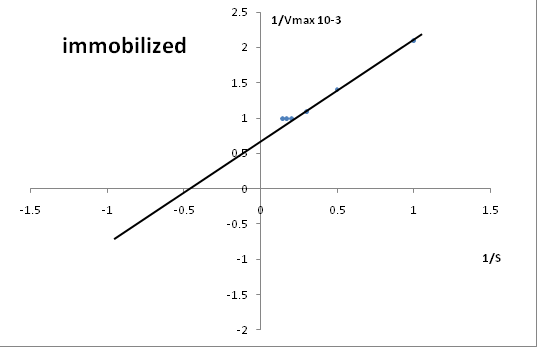


**Figure S1:** Estimation of kinetic parameters of the free and immobilized protease

**Table S1:** Effect of enzyme concentration on weight loss and the felting resistance of wool fibre using *Bacillus safensis* FO-36bMZ836779 [Bio-treatment conditions: 25% (v/v) TP enzyme, 55°C, pH 7, and liquor ratio: 1:50]

| **Conc.(%)/activity (unit)** | **Loss in weight (%)** | **Felt ball diameter (cm)** |
| --- | --- | --- |
| Untreated | -- | 2.12 |
| 5/1000 | 3.5 | 2.16 |
| 10/2000 | 6.9 | 2.24 |
| 25/5000 | 20.7 | No ball |
| 50/10000 | 24.2 | No ball |

Table S2: The change percent in the amino acid content of the bio-treated woolfibre relative to the untreated wool

| **Amino acid** | **%Change wool** |
| --- | --- |
| Aspartic acid | 34.9 |
| Glutamic acid | 12.1 |
| Serine | 11.1 |
| Glycine | 7.2 |
| Histidine | 8.6 |
| Arginine | 3.8 |
| Threonine | 25.9 |
| Alanine | 14.7 |
| Proline | 45.1 |
| Tyrosine | 3.97 |
| Valine | 1.0 |
| Methionine | 22.1 |
| Cystine | 9.6 |
| Isoleucine | 3.9 |
| Leucine | 0.8 |
| Phenylalanine | 45.3 |
| Lysine | 21.4 |
